# Supplementary material for: Development of the Australian Dietary Guidelines Adherence Tool (ADG-AT): A Food Matching Protocol
Source: Nutrients. 2025 Mar 19;17(6):1071. doi: 10.3390/nu17061071 (PMC11944852; doi:10.3390/nu17061071)
Supplement: Supplementary file 1 [file nutrients-17-01071-s001.zip › nutrients-3490779-supplementary.pdf]

Supplementary material

Table S1. Matching DQES foods to Australian Dietary Guidelines database entries.

**Single match, direct**	
DQES food name	Mango
Search term/s	"mango"
ADG database match	**Mango, peeled, raw**

**Single match, not further defined**	
DQES food name	Baked beans
Search term/s	“baked bean”
ADG database match	Baked beans, all flavours (except tomato sauce), reduced salt<br>Baked beans, canned in BBQ sauce, regular<br>Baked beans, canned in tomato & cheese sauce, regular<br>Baked beans, canned in tomato sauce, reduced salt<br>Baked beans, canned in tomato sauce, regular<br>Baked beans, canned in tomato sauce, with sausages, regular<br>**Baked beans, canned, not further defined**

**Multiple matches, average**	
DQES food name	Ham
Search term/s	“ham”
ADG database matches	**Ham, leg, lean**<br>**Ham, leg, lean & fat**<br>**Ham, leg, lean & fat, canned**<br>**Ham, shoulder, lean & fat**<br>**Ham, shoulder, lean & fat, canned**

Table S2. Calculation of discretionary serves, based on one serve equivalent to 600kJ.

DQES food name	Ham
AUSNUT database matches (kJ/100g)	467 (Ham, leg, lean)<br>598 (Ham, leg, lean & fat)<br>482 (Ham, leg, lean & fat, canned)<br>477 (Ham, shoulder, lean & fat)<br>509 (Ham, shoulder, lean & fat, canned)
Average kJ/100g	(467 + 598 + 482 + 477 + 509) / 5 = 506.6
Average discretionary serves (serves/100g)	506.6 kJ / 600 kJ = 0.8
